# Supplementary material for: Increased Adipose Tissue Expression of IL-23 Associates with Inflammatory Markers in People with High LDL Cholesterol
Source: Cells. 2022 Sep 29;11(19):3072. doi: 10.3390/cells11193072 (PMC9563604; doi:10.3390/cells11193072)
Supplement: Supplementary file 1 [file cells-11-03072-s001.zip › cells-1934087-supplementary.pdf]

NEGATIVE CONTROL

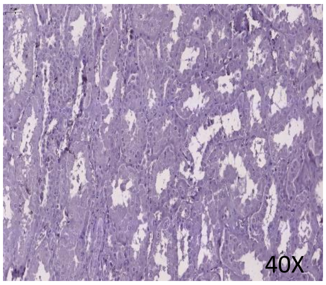

Isotype Rabbit IgG, polyclonal (Abcam® ab37415

POSTIVE CONTROL

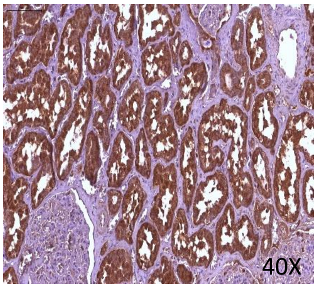

Rabbit polyclonal anti-IL-23 antibody (Abcam® ab115759)

Figure S1. Isotype Rabbit IgG, polyclonal (Abcam® ab37415) and rabbit polyclonal anti-IL-23 antibody (Abcam® ab115759) were used to perform immunohistochemistry (as described in the materials and methods) on the sections of human kidney tissue (positive control for IL-23.
